# PC5-Based Cellular-V2X Evolution and Deployment

**DOI:** 10.3390/s21030843

**Published:** 2021-01-27

**Authors:** Lili Miao, John Jethro Virtusio, Kai-Lung Hua

**Affiliations:** 1 Department of Computer Science and Information Engineering, National Taiwan University of Science and Technology, Taipei City 106, Taiwan; d10715005@mail.ntust.edu.tw (L.M.); d10715811@mail.ntust.edu.tw (J.J.V.); 2 Nokia Solutions and Networks, Taipei City 106, Taiwan

**Keywords:** C-V2X, autonomous driving, ITS, LTE-V2X, NR-V2X, RSU, OBU, SPAT, VRUCW

## Abstract

C-V2X (Cellular Vehicle-to-Everything) is a state-of-the-art wireless technology used in autonomous driving and intelligent transportation systems (ITS). This technology has extended the coverage and blind-spot detection of autonomous driving vehicles. Economically, C-V2X is much more cost-effective than the traditional sensors that are commonly used by autonomous driving vehicles. This cost-benefit makes it more practical in a large scale deployment. PC5-based C-V2X uses an RF (Radio Frequency) sidelink direct communication for low latency mission-critical vehicle sensor connectivity. Over the C-V2X radio communications, the autonomous driving vehicle’s sensor ability can now be largely enhanced to the distances as far as the network covers. In 2020, 5G is commercialized worldwide, and Taiwan is at the forefront. Operators and governments are keen to see its implications in people’s daily life brought by its low latency, high reliability, and high throughput. Autonomous driving class L3 (Conditional Automation) or L4 (Highly Automation) are good examples of 5G’s advanced applications. In these applications, the mobile networks with URLLC (Ultra-Reliable Low-Latency Communication) are perfectly demonstrated. Therefore, C-V2X evolution and 5G NR (New Radio) deployment coincide and form a new ecosystem. This ecosystem will change how people will drive and how transportation will be managed in the future. In this paper, the following topics are covered. Firstly, the benefits of C-V2X communication technology. Secondly, the standards of C-V2X and C-V2X applications for automotive road safety system which includes V2P/V2I/V2V/V2N, and artificial intelligence in VRU (Vulnerable Road User) detection, object recognition and movement prediction for collision warning and prevention. Thirdly, PC5-based C-V2X deployment status in global, especially in Taiwan. Lastly, current challenges and conclusions of C-V2X development.

## 1. Introduction

According to the statistics, 1.35 million people die in road accidents worldwide every year, equivalent to 3700 deaths per day [1]. Car crashes have risen to the 8th leading cause of death globally, and C-V2X (Cellular Vehicle-to-Everything) is becoming a popular solution to solve this problem [2]. C-V2X is a communication system that encompasses four sub-categories of applications [3]:

- **V2V (Vehicle-to-Vehicle):** between vehicle and vehicle, e.g., for keeping safe distance, speed, and lane changing.
- **V2I (Vehicle-to-Infrastructure):** between vehicle and road infrastructure, e.g., road sign, traffic light, toll gantry.
- **V2P (Vehicle-to-Pedestrian):** between vehicle and pedestrian, e.g., sensing nearby person or cyclist.
- **V2N (Vehicle-to-Network):** communication between vehicle and network, e.g., infotainment from the Internet, sending vehicle mechanical performance to its automaker.

C-V2X uses 3GPP The 3rd Generation Partnership Project) 4G (The fourth-generation) LTE (Long-Term Evolution) or 5G (The fifth generation) NR (New Radio) connectivity to transmit and receive signals. It uses two complementary transmission modes. The first is direct communications to vehicles, infrastructures, and road pedestrians. In this mode, C-V2X works independently of the cellular networks. It uses a PC5 interface for communication. The second mode is cellular network communications, in which C-V2X employs the conventional mobile network to enable vehicles to receive information about road and traffic conditions in the area. It uses Uu interface for communication.

With the evolution and deployment of C-V2X technology, fatal accidents due to human errors or road conditions and massive traffic jams due to special occasions or accidents will no longer be a problem. Detecting risks by C-V2X V2V and V2P before they become a threat and noticing traffic jams by C-V2X V2I and V2N before you see them will be coming soon. This vision of safer roads and more efficient travel can be realized by C-V2X, Intelligent Transport Systems, and 5G together.

In this article, we first describe the benefits of C-V2X and why it has been gaining popularity in recent years. We also describe the specifications of C-V2X in LTE and NR defined by 3GPP and introduce a few application scenarios to show that C-V2X can provide safer roads, more efficient travel, and better driving experiences. Moreover, we investigate worldwide C-V2X deployment progress. This work contains many technical research works and insights from a C-V2X technical expert from Nokia who designed a series of workable system integration solutions that have been deployed in some pilot and commercial C-V2X projects. This pioneering study discusses C-V2X technology applied in practice, and all the test results and conclusions come from real C-V2X communication system integration, not simulation tools. These projects are to be introduced one by one in a later chapter. Finally, the C-V2X challenges in the current ecosystem are discussed. We expect that through this work, we can promote C-V2X technology and take it forward.

## 2. C-V2X Benefits

C-V2X with low latency and high-reliability allows vehicles to communicate with other vehicles (V2V), pedestrians (V2P), roadside infrastructure (V2I), and networks (V2N) with or without a cellular network [3] and address road safety and traffic efficiency. Typically, autonomous driving vehicles are equipped with advanced sensors: Camera, LiDAR (Light Detection and Ranging), Radar, GNSS (Global Navigation System Satellite), and CAN (Controller Area Network). So why would it still be beneficial for intelligent transportation systems to adopt C-V2X technology? It is because C-V2X can detect potential hazards and road situations from longer distances. A fully equipped autonomous driving vehicle cannot detect non-line-of-sight (NLOS) objects. C-V2X can overcome NLOS problems by using PC5 interface sidelink communication or cellular network for additional safety features. Sensors in the vehicle provide the basic functionalities of autonomous driving; this will not change in the future and is crucial for safety. But the automotive industry, however, has recognized that connectivity is necessary to increase the safety and comfort of L3 (Level 3: Conditional Automation) or L4 (Level 4: Highly Automation) driving further. Going beyond a certain level of driving autonomy will require vehicles to be connected with C-V2X [4].

**Figure 1:**
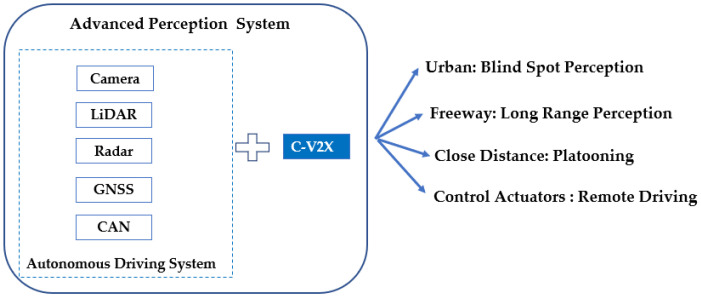
C-V2X Benefits.

C-V2X can aggregate information collected in the cooperative perception, update the map with exact road structure information, and distribute the localized HD (High Definition) map to vehicles based on their locations. These enhanced and advanced services such as blind-spot detection, long-range perception, remote driving and platooning, etc. C-V2X can increase road capacity, driver safety, and comfort. All the C-V2X benefits for autonomous driving are summarized in Figure 1.

Early V2X technologies, like DSRC (Dedicated Short Range Communication) in the USA and ETSI (European Telecommunications Standards Institute) ITS-G5 in Europe, are based on IEEE (Institute of Electrical and Electronics Engineers) 802.11p, which adds wireless access in vehicular environments (WAVE) at the physical layer and the medium access control (MAC) layer. Currently, 802.11bd is under development as the next-generation standard. DSRC has since been used in many countries for V2V, V2P, and V2I applications. But it has not been commercialized on a large scale for almost two decades [2]. Compared with C-V2X, DSRC has many limitations [5]: (1) Its protocol algorithm CSMA-CD (Carrier Sense Multiple Access with Collision Detection) for V2V and V2I direct communications have hidden nodes, data competition, and collision problems; (2) Its visible and non-visible transmission distances are very limited; (3) It is unable to integrate with the existing cellular mobile network. Hence, in recent years, global V2X development is towards using cellular technology C-V2X, which has high performance and a forward evolutionary path towards 5G, to support terminal mobility such as seamless handover and roaming abilities. USA federal government agency NHTSA (National Highway Traffic Safety Administration) expressed its favor for C-V2X [6]. Furthermore, FCC (Federal Communications Commission) has recently announced its preference of C-V2X over DSRC for ITS (Intelligent Transport Systems) radio service [2].

**Figure 2:**
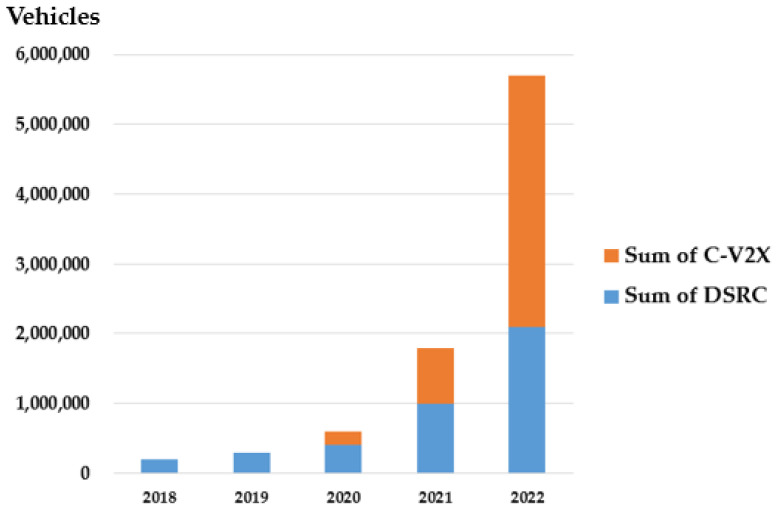
Worldwide forecast for the number of vehicles equipped with V2X technology [7].

Worldwide forecast for the number of vehicles equipped with V2X technology is shown [7] in Figure 2.

It is worth mentioning that DSRC and C-V2X technologies are addressing similar use-cases. They have an identical network, security, and application layers; the only difference is in the access layer. Therefore, some companies [8,9] are delivering dual-mode functionality device to support both DSRC and C-V2X technologies. Technology is meaningful only when it can serve humans. It is yet to be seen which one will the market prefer.

## 3. C-V2X Standard and Application Scenarios

**Figure 3:**
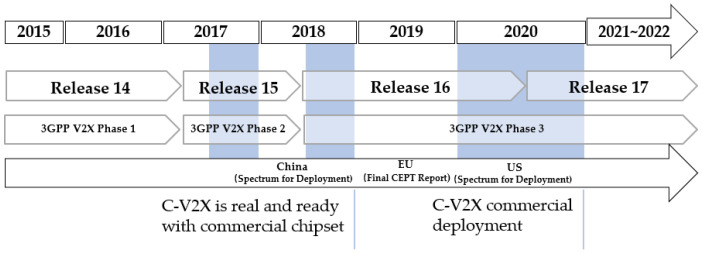
C-V2X 3GPP standard and deployment milestone [10].

The evolution of C-V2X standardization in the 3rd Generation Partnership Project (3GPP) and deployment milestone in 5G Automotive Association (5GAA) [10] with chipset availability are summarized in Figure 3.

### 3.1. LTE-V2X

Completed in March 2017, phase 1 of 3GPP Rel-14 provided the initial standards supporting V2V services and the additional V2X services leveraging the cellular infrastructure. The primary safety features of C-V2X under 3GPP Rel-14 were delivered through cellular networks or sidelink communications via the PC5 interface. A new LTE (Long-Term Evolution)-V2X band 47 (with 10 and 20 MHz bandwidth) was introduced to support C-V2X by unlicensed 5.9 GHz spectrum. 3GPP Rel-14 also introduced two new physical channels for PC5-based C-V2X communication, namely the Physical Sidelink Shared Channel (PSSCH) and Physical Sideline Control Channel (PSCCH). As the name discloses, PSSCH carries data, and PSCCH has control information for decoding the data channel in the physical access layer. To accelerate the LTE-V2X development, LTE-V2X uses the centralized scheduling mode (Mode 3), and decentralized scheduling mode (Mode 4) [11] of LTE-D2D (Device-to-Device), supporting sidelink communications via PC5 interface. The cellular network allocates resources under Mode 3. On the other hand, Mode 4 does not require cellular coverage; vehicles can autonomously select their radio resources using a sensing-based Semi-Persistent Scheduling (SPS) scheme supported by congestion control mechanisms. In June 2018, 3GPP Rel-15 was completed. The enhanced V2X services such as platooning, extended sensors, advanced driving, and remote driving [12] were introduced as phase 2 of 3GPP V2X, which has formed a stable and robust ecosystem around LTE-V2X:

- **Platooning:** Vehicles dynamically form a platoon traveling together. All vehicles in the platoon exchange information that enables them to maintain a short distance safely.
- **Extended Sensors**: Exchange of raw or processed sensor data among vehicles, roadside units, devices of pedestrians, and V2X application servers, enhancing environment perception beyond what sensors can detect (e.g., via the exchange of live video).
- **Advanced Driving**: Enable semi-automated or fully-automated driving. Perception data obtained from local sensors and driving intention exchanged with vehicles in proximity to synchronize and coordinate.
- **Remote Driving**: Remote driver or V2X application operates remote vehicle (e.g., for disabled passenger, vehicle in dangerous environment, predictable route driving, etc.).

### 3.2. NR-V2X

**Figure 4:**
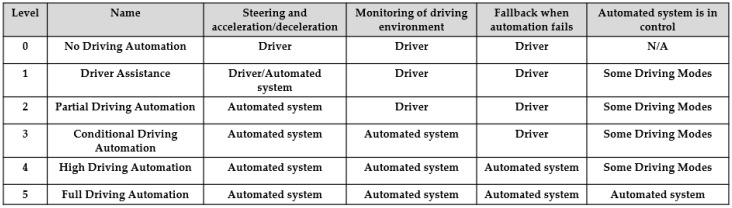
The 5 levels of driving automation [13].

5G NR (New Radio)-V2X as phase 3 of 3GPP V2X is backward compatible at upper layers of LTE-V2X. To meet the low latency and reliability requirements for the advanced V2X services, NR-V2X is designed to support these applications. As a V2N type application, 5G URLLC (Ultra-Reliable Low-Latency Communication) network slicing can provide autonomous driving advanced functions for L3 (Conditional Automation) and L4 (Highly Automation) of Figure 4 [13] with higher QoS (Quality of Service) profile.

Some of these advanced application scenarios require the transmission of periodic traffic. So besides broadcast, two new communication types, unicast and groupcast, are introduced in 5G NR-V2X. Similar to LTE-V2X, 5G NR-V2X [14] also defines two types of sidelink communication modes: Mode1 and Mode2. The NR-V2X Mode 1 defines the mechanisms that allow direct vehicular communications while the cellular network base station allocates radio resources to the vehicle by the Uu interface. Mode 2 supports direct vehicular communications via PC5 interface under the out-of-coverage of the cellular network. 3GPP Rel-16 has been officially frozen in July 2020 [15]. In the developing of 3GPP NR Release 17 [16], new sidelink communication relaying architecture is proposed to support some of the advanced V2X services. The difference between LTE-V2X and NR-V2X is highlighted in Table 1.

Since PC5-based C-V2X Mode4 [11] does not require a cellular network, normally two kinds of wireless devices **RSU (Road Side Unit)** and **OBU (On Board Unit)** are sufficient to deploy C-V2X V2I/V2V/V2P application scenarios:

1. **RSU**: Wireless transmitting device can provide direct sildelink communication via PC5 interface without cellular networks. Road signs, traffic lights, and IP camera information can be broadcasted in real-time to the vehicle via RSU in the predefined area. Another useful scenario is that RSU can be equipped with a SIM card to transfer road information via cellular networks to create additional applications for public safety.
2. **OBU**: Installed in a vehicle as a wireless communication device to enhance autonomous driving vehicles’ sensor function by direct communication with RSU and other OBUs. OBU is responsible for broadcasting vehicle’s location, direction, and speed information to other defined devices while receiving other vehicle’s data as input for its internal algorithms to avoid possible accidents.

### 3.3. PC5-Based C-V2X Application Scenarios

To utilize C-V2X applications, RSU/OBU devices must be equipped with 3GPP C-V2X [3] compliant chipsets. Commercial availability of such chipsets [10] is from Qualcomm, Intel, Huawei, Datang, Autotalks. Currently, Taiwan uses Qualcomm 9150 for PC5-based C-V2X field testing and commercial deployments. Many exciting applications [12,18,19,20] have been realized. Some application scenarios are highlighted below:

- **SPAT (Signal Phase and Timing Message)**: a V2I service that integrates traffic signal controller (light color and remaining time) with long-range wireless transmission device RSU, which broadcasts them to the OBU. Depending on this information, the driver or autonomous driving control unit can decide whether to change the route or speed up.
- **TSP (Traffic Signal Preemption)**: a V2I service that allows high-priority vehicles, such as ambulances, fire trucks, and police cars approaching the signal control intersection to send a priority signal so that the vehicles can pass through.
- **VRUCW (Vulnerable Road User Collision Warning)**: a V2P service that alerts the driver or autonomous driving control unit when a vehicle is sensing a potential pedestrian collision threat via IP Camera and RSU on the roadside.
- **ICW (Intersection Collision Warning)**: a V2V service that warns the host vehicle driving towards an intersection if there is a risk of collision.
- **EBW (Emergency Brake Warning)**: another V2V service, which warns the host vehicle if the front remote vehicle is making a sudden brake. The host vehicle receives an alert from the front vehicle and determines if a collision will happen.
- **DNPW (Do Not Pass Warning)**: a V2V service used when a host vehicle is planning to overtake the front vehicle via opposite direction lane. The host vehicle will alert the nearby vehicle traveling in the opposite direction. The host vehicle’s OBU will receive a DNPW message to decide if it is safe to overtake.
- **HLW (Hazardous Location Warning)**: a V2I service that warns a host vehicle of potentially hazardous situations, such as deep waters after a storm, deep pits on the road surface, or slippery roads.

All the above-described application scenarios can be deployed by using PC5-based C-V2X direct communication technology. However, the 4G LTE cellular network can’t support advanced C-V2X applications due to limited performance. 5G NR has opened an opportunity for time-sensitive applications. The future is inspiring for C-V2X once full 5G coverage is available.

## 4. C-V2X Deployment in Global

Even 802.11p based V2X technology was early adopted by some global automotive manufacturers as an electronic toll collection technology because C-V2X demonstrates a forward evolutionary path towards 5G. 5GAA [10] started to develop C-V2X technology. Moreover, FCC (Federal Communications Commission) officially announced to allocate 5.9 GHz ITS spectrum for C-V2X in December 2019 [21]. Finally, FCC decided to retain 30 megahertz of spectrum in the 5.895–5.925 GHz for the ITS radio services using C-V2X technology in November 2020 [2]. Meanwhile, Europe is developing a new EN (European Standard) to define the use of C-V2X as an access layer technology for C-ITS (Cooperative-Intelligent Transport System), which is approved by ETSI. Australia originally started on-road tests of C-V2X technology in Victoria, late 2018. In China [10,14], first C-V2X trials were launched in 2016 by using Triple-level CATT(Datang), Huawei Hisilicon and Qualcomm chipset. Interoperability testing of PC5-based LTE-V2X applications with Multi-vendor Interoperability had finished in Shanghai in November 2018, and C-V2X “Four Layers” Interoperability Application Demonstration focusing on security mechanism was organized in Shanghai in October 2019. Japan C-V2X trial began in 2018, and the use cases include V2V, V2P, V2I, as well as V2N operations over cellular network-based wide area communications with cloud access. South Korea had successfully demonstrated 5G C-V2X communications between autonomous test vehicles (AVs) in 2019.

**Figure 5:**
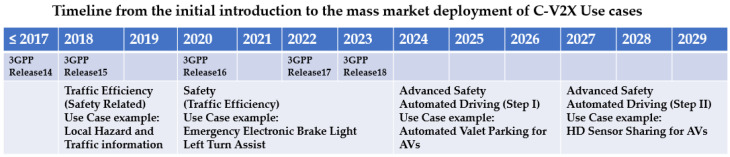
Timeline from the initial introduction to the mass market deployment of C-V2X Use cases [22].

Considering the current 3GPP releases and supply chain readiness, 5GAA even developed a long-term blueprint of traffic efficiency and basic safety C-V2X use cases worldwide in September of 2020 [22] as shown in Figure 5.

As a result, C-V2X has gained momentum in the United States, Europe, Australia, China, Japan, Korea, and other markets. C-V2X is dominating globally, and Taiwan is one of the countries at the forefront of C-V2X development supported by the government in the intelligent transportation systems programs [23].

## 5. PC5-Based C-V2X Deployment in Taiwan

In this chapter, some Taiwan field tests and commercial projects, whose C-V2X networks are implemented by network solution provider Nokia, are introduced. As a C-V2X technical expert, the author from Nokia provides a sequence of PC5-based C-V2X system integration solutions. (1) Translating traffic light control signaling into C-V2X internal massages recognized by RSU/OBU to realize SPAT application. Normally, autonomous driving vehicles are equipped with cameras and artificial intelligence that can recognize traffic light information. However, recognition accuracy is easily affected by bad weather or occlusions. Our proposed solution is designed to be more robust against any conditions that can hinder visual recognition. (2) Utilizing AI technology, which has shown state-of-the-art performance in many problem domains [24,25,26], for VRUCW application. Deep Learning-based Vulnerable Road User Detection and Collision Warning can be provided by PC5-based C-V2X system architecture. (3) Integrating C-V2X to Automated Driving Systems (ADS) for enhanced safety. By this solution, ADS can monitor road conditions, detect potential problems, and take action to avoid road accidents. The success of these projects will lay a solid foundation for the coming 5G NR-V2X.

**Figure 6:**
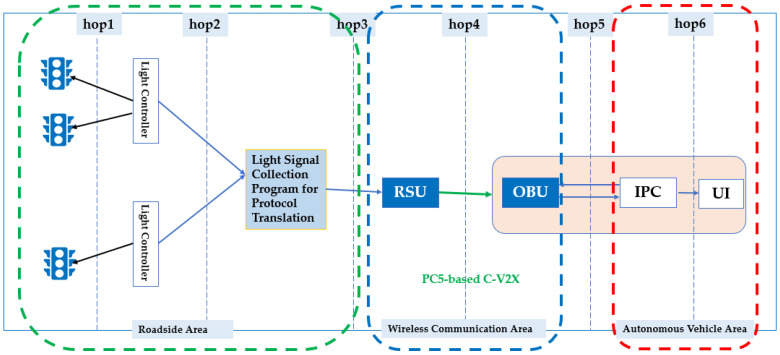
C-V2X SPAT Application System Architecture.

**Figure 7:**
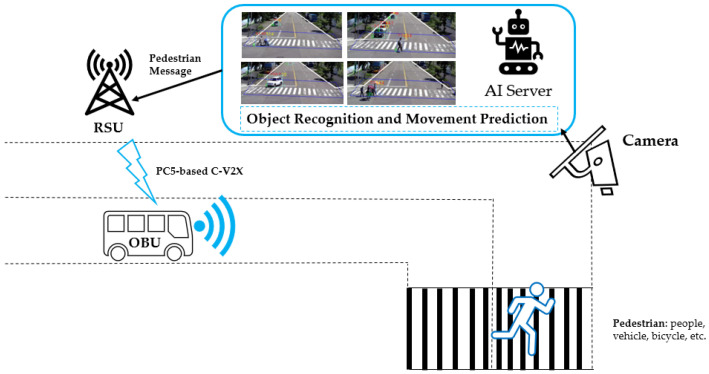
PC5-based C-V2X VRUCW.

- **Solution1: Traffic Light Control System Integration**
  To realize SPAT application locally, we have designed a system architecture shown in Figure 6. Accordingly, the PC5-based C-V2X SPAT application has been launched successfully and is introduced in this chapter.
  1. We can collect traffic light information from the light controller directly.
  2. Light signaling collection program is responsible for receiving roadside light signal information, including light phase, color and remaining time, etc. All this information is sent to RSU.
  3. RSU reads and encapsulates them to C-V2X protocol messages.
  4. RSU broadcasts C-V2X messages to OBU via PC5 interface.
  5. OBU installed in the autonomous driving vehicle analyzes and filters these messages and then sends them to IPC (Industrial PC) of autonomous driving system for slow or stop control.
  6. User Interface (UI) displays light information for intuitive C-V2X technology presentation.
- **Solution2: C-V2X VRUCW Application System Integration**
  For the PC5-based C-V2X VRUCW application, we have developed a network diagram shown in Figure 7.
  1. The VRUCW application can be regarded as P2I2V service ( Pedestrian to Infrastructure to Vehicle). The IP cameras must be built on road areas for both line-of-sight (LOS) and non-line-of-sight (NLOS) monitoring purposes.
  2. It uses an AI server equipped with a series of deep learning technologies such as CNN (Convolutional Neural Network) and SSD (Single Shot Detection). Objects will be detected if any pedestrian passes an area under camera coverage.
  3. The AI server transfers the analysis result with object recognition and movement prediction to RSU, which broadcasts this information to all the OBUs under the coverage of this RSU.
  4. OBU is responsible for combining vehicle information such as speed, heading, and location to determine if a collision is approaching. We use the target classification algorithm to determine the pedestrian’s direction for subsequent warning possibility calculation.
  5. Supposing that there is a risk of collision between pedestrian and host vehicle, for example, their distance is within 50 meters and driving speed of vehicle is more than 10km/hour. We use below Algorithm 1 for collision warning trigger.

**Definition** **1.** *$Head_{h}$: Azimuth angle of the current host vehicle in position point H. $Head_{n}$: Azimuth angle of the target pedestrian in position point N. $\left(-180^{\circ },180^{\circ }\right)$: The range of azimuth angle for $Head_{h}$ and $Head_{n}$. $20^{\circ }$: Compare Threshold*

**Algorithm 1**
 **if**
$\left|Head_{n}-Head_{h}\right|<20^{\circ }$
**then**  the target pedestrian is heading in the same direction as the host vehicle, no collision warning is sent **else if**
$\left|Head_{n}-Head_{h}\right|>160^{\circ }$
**then**  the target pedestrian is heading in the conflict direction with the host vehicle, collision warning is sent **else if**
$20^{\circ }<\left|Head_{n}-Head_{h}\right|<160^{\circ }$
**then**  the target pedestrian is crossing on the left side of the host vehicle, collision warning is sent **else if**
$-160^{\circ }<\left|Head_{n}-Head_{h}\right|<-20^{\circ }$
**then**  the target pedestrian is crossing on the right side of the host vehicle, collision warning is sent **end if**

**Figure 8:**
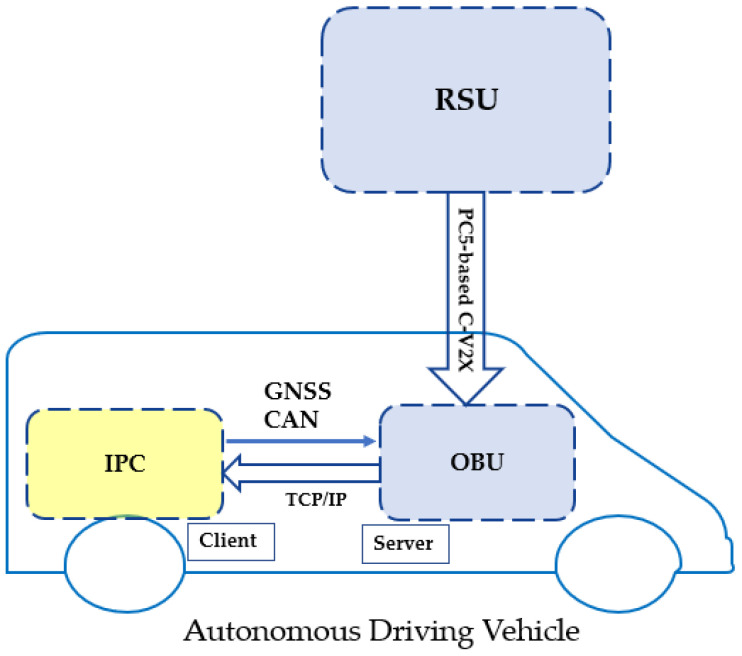
C-V2X Automated Driving System Integration.

- **Solution3: Automated Driving System Integration**
  We have designed and implemented the system integration between PC5-based C-V2X and automated driving system, shown below in Figure 8:
  1. RSU receives information from traffic light controllers or AI servers. Then, it broadcasts this information by using a predefined message format in its coverage field.
  2. OBU receives broadcasting messages via PC5-based C-V2X communication.
  3. OBU is connected with IPC (Industrial PC) of the autonomous driving system via TCP/IP. OBU receives GSNN (Global Navigation System Satellite) and CAN (Controller Area Network) messages from the vehicle.
  4. OBU uses advanced internal algorithms to judge if the situation is dangerous or not. Then a warning message will be sent accordingly to the IPC of the autonomous driving system.
  5. Finally, C-V2X technology is integrated with the automated driving system as expected.

Based on the above solutions that were discussed, the following chapters will introduce which projects are implemented.

### 5.1. V2V and V2N Field Testing

**Figure 9:**
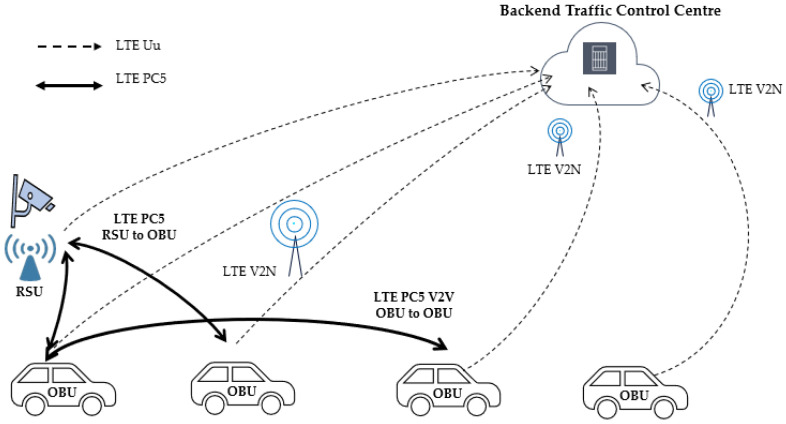
C-V2X Network Topology.

Cellular infrastructure provider Nokia and its Partner research institutes in Hsinchu have been in cooperation to test C-V2X since 2018. Our contribution is to integrate all the systems with the most advanced technologies to prove C-V2X technology workable. The test involves autonomous driving vehicles RSU/OBU, which were both provided by Nokia. RSU and OBU were developed based on Qualcomm 9150 Chipset, which gives optimum performance. PC5-based C-V2X network topology is showed in Figure 9.

The test steps are described below:

1. One RSU is installed to extend the test vehicle’s field of vision. The RSU transmits predefined road information such as pedestrian detection to OBU by PC5-based C-V2X.
2. OBUs are installed in the test vehicles to receive RSU information as well as between OBUs via PC5-based V2V application.
3. RSU/OBU information is transferred back to “Traffic Control Centre” by 4G LTE cellular network as a V2N application.

**Figure 10:**
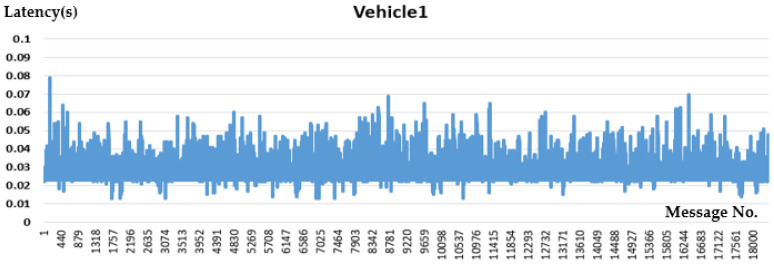
Vehicle1 Performance.

**Figure 11:**
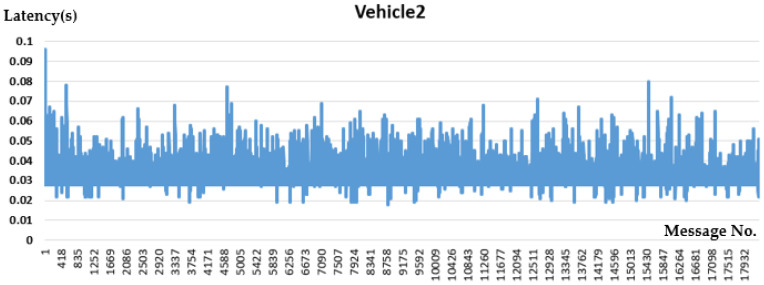
Vehicle2 Performance.

V2V PC5 interface communication performance is evaluated below in Figure 10 and Figure 11.

From the V2V test result in Table 2, the average latency is 30 ms, which indicates that the device involved with PC5-based C-V2X has better performance than the Rel-14 standard defined maximum latency of 100 ms for V2X applications.

### 5.2. Commercial Pilot

This project [27] is initiated by the New Taipei City government under the city intelligent transport program. PC5-based C-V2X technology has been applied in an open road environment for the first time in Taiwan. The city cooperates with the Ministry of Transport to use an autonomous driving bus in Tamsui Town as an extension of the MRT Danshui line. After six months of system stability testing, the project has been in service since August 2020 [28]. The public is given free rides to experience the new technology. The New Taipei City government expects to make people more interested in the “Smart Driving Bus” concept.

### 5.3. Commercial Project

A commercial C-V2X project [29] is implemented in Chang Gung Health And Culture Village as part of its technology transformation strategy. This project promotes road safety under 5G use cases for the elderly and their caregivers. The PC5-based C-V2X is used for VRUCW (Venerable Road User Collision Warning) services to provide convenience and extra protection for the aged residents and their caretakers in the village. Once the 5G NR cellular network is ready, more C-V2X services such as SPAT, remote driving management can be implemented to build up a highly collaborative and integrated ecosystem of “People-Vehicle-Road-Network-Cloud” five dimensions.

## 6. Discussion

C-V2X can improve road safety, traffic efficiency, and distribution of road information. It is relatively low cost and highly effective compared to traditional onboard sensors. Standardization of LTE-V2X and NR-V2X has been actively promoted by 3GPP, which has motivated many organizations to develop C-V2X technology. However, there are still some challenges in PC5-based C-V2X deployment.

- C-V2X is an eco-system. There must be robust engagements from industry stakeholders, like road transport authority, autonomous driving developers, network transmission operators, and government. To improve C-V2X, the government needs to push road transport facility development and unify its standards. For example, traffic light control systems need to be upgraded from traditional equipment to higher processing devices. To transmit the traffic information promptly, it requires a traffic light control system to send signal changing information with a predefined frequency of at least 10HZ. But existing devices across Taiwan can’t meet this requirement. A mediation conversion process must be added. The disadvantage of this process is that it increases the message transport latency. So, there are delays between the traffic light control console and traffic lights, which is against the ITS standard. This problem causes difficulties in obtaining the correct timing information for C-V2X devices to synchronize in the SPAT application. To overcome these problems, the government must develop a unified standard to push the upgrade of traffic light control systems.
- Technical application layer specifications are not standardized in C-V2X. Some organizations follow up European Standard, while some use American Standard or combine both as a national standard. It is still unclear which standard will be adopted globally. Unifying and balancing all the pros and cons should be part of the government’s smart city agenda.
- In some regions such as Taiwan, 5G is still a year away from island-wide coverage, though it has completed LTE-V2X service testing and trials. So far, it is only focused on applications with limited KPI (Key Performance Indicator) performance. C-V2X will reach a new level once 5G is introduced, where bandwidth, low latency, and high throughput are critical for the use case. However, the deployment of 5G NR-V2X still needs the entire eco-system to be fully adopted.
- Plenty of privacy and confidential information from vehicles, roadside infrastructure, and IP cameras are broadcast in the public space. Protecting information security is an essential topic for PC5-based C-V2X deployment, as mentioned in [30,31,32]. The country needs a standard to define a security policy.
- Autonomous Driving is defined as Level 0–Level 5 according to international specification SAE J3016 [13]. However, there are no international standards for road level definition.
- Regulations and insurance claims for ITS road accident are yet to be defined.

## 7. Conclusions

In this work, we have introduced the standardization of C-V2X in 3GPP. The critical difference between LTE-V2X mode 3 and mode 4, NR-V2X mode 1 and mode 2 are discussed. Operators and governments around the world are excited to see 5G technology applied in C-V2X. Countries with early adoption of LTE-V2X are certainly at the forefront. As our contribution, we have proposed some workable solutions for ITS, AI, and ADS integration. In our lives today, digitization is everywhere, and people need what 5G provides now more than ever. Our purpose in this article is to verify C-V2X technology feasibility and accessibility as proof of concept, then revise technology flaws to accumulate academic improvement. The field tests have been done to conclude that the technology is promising. The success of the field tests illustrated in this paper will motivate manufacturers to invest and the government to define favorable legislation towards the development of C-V2X. Taiwan is a world-leading electronic manufacturer and is in an advantageous position to develop intelligent transportation systems and utilize autonomous driving with C-V2X technology. However, there are still some research challenges and limitations that must be addressed. For instance, C-V2X deployment requires the cooperation of different industries. A nationwide C-V2X network requires millions of RSUs and OBUs. Full 5G coverage in the country isn’t available yet. Our future research direction includes network coverage, latency, reliability, and security policies. Because many of the V2I, V2V, and V2P applications are mission-critical, any gaps or glitches would mean life or death. 

## Figures and Tables

**Table 1:** Difference between LTE-V2X and NR-V2X.

Items	LTE-V2X	NR-V2X
Specification	3GPP Rel-14/Rel-15	3GPP Rel-16/Rel-17
Latency	Low latency: 10~100 ms	Ultra-low latency: 1 ms [17]
PC5 Message Type	Broadcast	Broadcast, Unicast and Groupcast
Application Scenario	Safety related/Enhanced	Advanced Application
Cellular Network Coverage	Uu and PC5 Mode3	Uu and PC5 Mode1
Out of Cellular Network Coverage	PC5 Mode4	PC5 Mode2

**Table 2:** V2V Test Result.

Performance (between OBUs)	Vehicle 1	Vehicle 2
Packet Transmission Frequency	100 Hz	100 Hz
Average Latency	27 ms	32 ms
Package Drop Rate	2.47%	2.72%

## Data Availability

Section 5.1Table 2: https://drive.google.com/file/d/1Nu2umEjbBYQpswGZm_XPaTuns2fGs-YP/view?usp=sharing.
